# Newly designed plate for the treatment of posterolateral tibial plateau fractures: a finite element analysis

**DOI:** 10.1186/s13018-024-04686-z

**Published:** 2024-03-26

**Authors:** Zhaokui Yan, Chang Zou, Guy Romeo Kenmegne, Xuelin Pan, Nawin Ghimire, Kuruwitage Manthi Nihara Silva, Yue Fang

**Affiliations:** 1 Department of Orthopedics, West China Hospital of Sichuan University, Chengdu, 610041 China; 2 Trauma center, West China Hospital of Sichuan University, Chengdu, 610041 China; 3 Department of Radiology, West China Hospital of Sichuan University, Chengdu, 610041 China

**Keywords:** Posterolateral, Tibial plateau fracture, New plate, Internal fixation, Finite-element analysis, Osteosynthesis

## Abstract

**Background:**

This study investigated the biomechanical properties of a new plate used for the treatment of posterolateral tibial plateau fractures using finite element analysis.

**Methods:**

The study groups were as follows: group PM, model of the new plate with posteromedial tibial plateau fracture; group PL, model of the new plate with posterolateral tibial plateau fracture; and group PC, model of the new plate with posterior tibial plateau fracture. We used two loading modes: uniform loading on the entire plateau, and loading on the posterior plateau. Data such as the displacement of the fracture and distribution of stress on the new plate and screws were recorded and analyzed.

**Results:**

When the whole plateau was loaded, the displacement of fractures in groups PM, PL, and PC were 0.273, 0.114, and 0.265 mm, respectively. The maximum stresses on the plates in groups PM, PL, and PC were 118.131 MPa, 44.191 MPa, and 115.433 MPa. The maximum stresses on the screws in Groups PM, PL, and PC were 166.731, 80.330, and 164.439 MPa, respectively. When the posterior tibial plateau was loaded, the displacement of the fractures in groups PM, PL, and PC was 0.410, 0.213, and 0.390 mm, respectively. The maximum stresses on the plates in groups PM, PL, and PC were 194.012 MPa, 72.806 MPa, and 185.535 MPa. The maximum stresses on the screws in Groups PM, PL, and PC were 278.265, 114.839, and 266.396 MPa, respectively.

**Conclusion:**

The results of this study revealed that titanium plates have good fixation effects in all groups; therefore, the use of the new plate for posterolateral tibial plateau fractures appears to be safe and valid.

## Introduction

Posterolateral tibial plateau fractures have been frequently discussed by trauma specialists in recent decades [1, 2]. The principles of surgical treatment for tibial plateau fractures include anatomic reduction, strong internal fixation, and bone grafting, if necessary, while maintaining the normal mechanical axis of the lower extremity.

At present, there are some reported studies on the internal fixation of posterior tibial plateau fractures [3]. Hu et al. [4] used a pre-bending T-shaped distal radius plate and rafting screws to treat 12 cases of isolated posterolateral tibial plateau fractures, and the HSS score reported was 93.2 points at 1 year follow up after the operation. Yu et al. [5] used an annular plate to treat 22 cases of type II-VI lateral tibial plateau fractures, and the HSS score reported by the authors was 82–95 after a follow-up of 13–32 months. In another study, posterolateral tibial plateau fractures were treated with different fixation methods, such as lateral + posterolateral locking plate fixation, lateral + posterolateral locking plate fixation + 1/4 tubular plate edge fixation, and [6]. Ren et al. [7] developed a new curved support plate (CSP) to investigate the treatment modalities for posterolateral tibial plateau fractures using a traditional anterolateral approach. Other scholars, through osteotomy of the lateral femoral epicondyle combined with osteosynthesis using a one-third tubular horizontal belt plate, treated lateral depressions of the posterolateral tibial plateau [8].These studies carry important clinical values; However, due to the irregular anatomy of the bone structure behind the tibial plateau, there is a lack of special posterior support plate that can be widely recognized and used to treat the posterolateral tibial plateau fractures. Therefore, a combination of one or more pieces of reconstruction or radial plates is used to treat posterolateral tibial plateau fractures by means of pre-bending and truncation, which increases the surgical time. Such plate and technical variability among surgeons can lead to weak fixation, which can cause traumatic arthritis and consequent knee dysfunction [9, 10].

Therefore, we developed a new plate specifically designed for the treatment of posterolateral tibial plateau fractures. The new plate simulates the normal anatomical deviation of the posterior tibial plateau, making it more attached to the bone surface and thus providing better attachment and support. To provide guidance for future clinical applications, the biomechanical characteristics were explored using finite element analysis.

## Materials and methods

The experimental devices were an HP820 server (inter E5-2690) and two processors (each containing eight cores, 16 threads, and 48G memory). The modeling software HyperMesh 2017, calculation software Abaqus 6.12, and post-processing software hyper view 2017 were used in the experiment; a copyright license was obtained from the above mentioned software owners. The anatomical parameters of the new plate design were derived from the normal tibial CT data at our hospital’s imaging center. For the purpose of this study, data were collected from a pool of 50 adult Chinese patients, out of which an adult male tibia was selected to construct a CAD model. The model used in this experiment was extracted from the CT database of West China Hospital; the study was approved by the Biomedical Ethics Review committee of West China hospital, Sichuan University and the informed consent was obtained from all involved participants. The bone shape of a specific population was obtained using the mimic software (copyright License obtained), and the bone shape was extracted by smoothing and removing spikes. A CAD model of the plate was constructed using Unigraphics NX(UG), and the hole shape was added to obtain the final nail plate model.

We selected data from the patients with unilateral tibial plateau fractures. The data for the affected limb were used to establish the CAD model of the tibial plateau fracture, whereas the data for the uninjured limb were used to establish the normal tibial plateau CAD model. According to Luo et al., [11] patients were categorized as Group PM (posterior + lateral column fracture), Group PL (posterior + medial column fracture), and Group PC (double column + posterior column fracture); when the modified Schatzker classification [12] was considered, they could be arranged as follows: Group PM, IIP; Group PL, IVP; and Group PC, VP.

The implants, including plates and screws, were stimulated at Ti6Al4V. Both the implants and bone were assumed to be elastic, linear, and composed of isotropic materials. The elastic moduli of the cortical bone, cancellous bone, and implant were 8844 MPa, 660 MPa, and 110,000 MPa, respectively, and Poisson’s ratios were 0.26, 0.0.20, and 0.33, respectively). Table 1 lists the material properties with corresponding references [13, 14].

**Table 1:** Materials and machined alloy composition

Material	Machined Alloy composition (mass %).						
**Alloy**	C	Fe	N	O	Al	V	Ti
Ti6Al4V	0.08	0.164	0.05	0.05	5.47	4.09	Rest
**Material**	**Elastic modulus(MPa)**			**Poisson’s ratio**			
Titanium (Ti6Al4V)	110,000			0.3			

*Note* C = Copper, Fe = Iron, N = Nitrogen, O = Oxygen, Al = Aluminium, V = Vanadium, Ti = Titanium

### Creating a CAD model

**Fig. 1:**
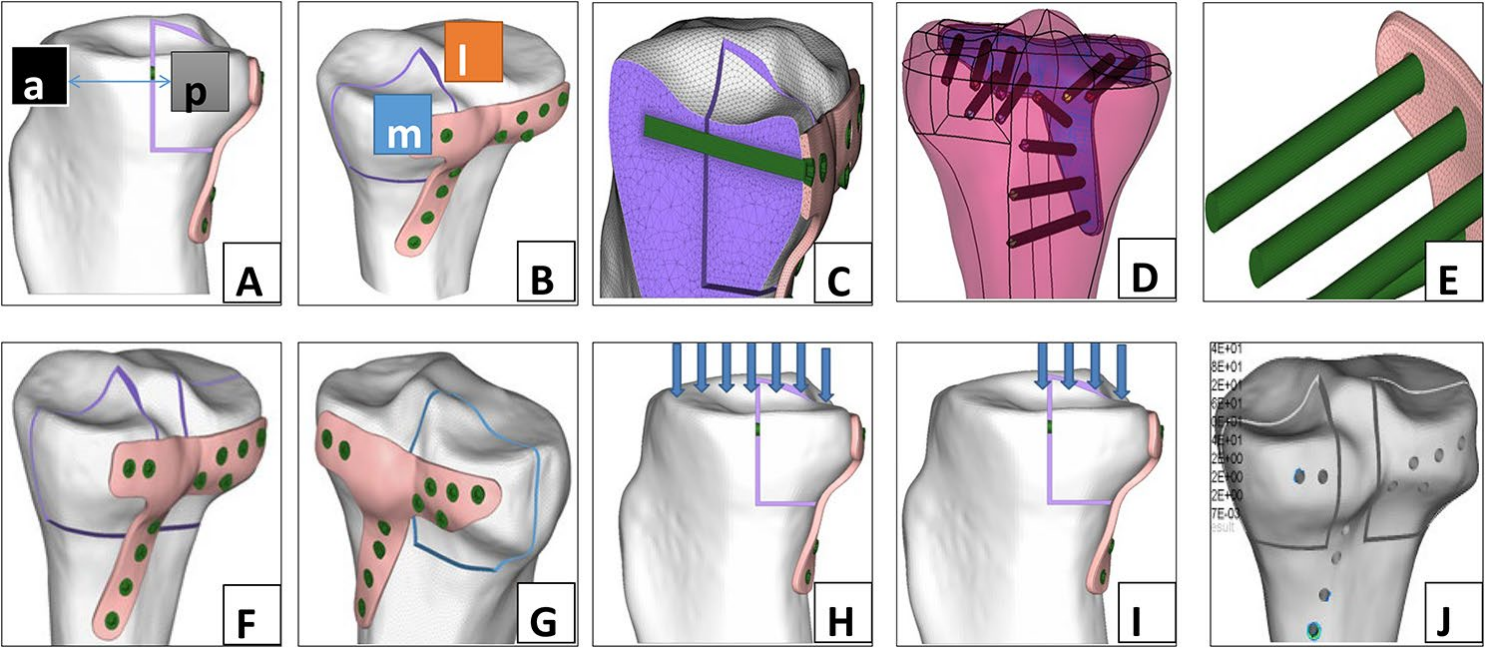
CAD model of the tibial plateau fracture: the distal end of the fragment was cut completely to separate the fragment from the tibial plateau medial view (**A**) and posteromedial view **(B**), the inner and outer structures of bone exposed (**C**), the model, plate and screw fixed (**D**), plate and nail cap tied simulating locking system and (**E**), Two sides of tibial plateau fully presented; medial side (**F**) and lateral side (**G**), forces loaded uniformly on the tibial plateau on the full plateau (**H**) and on the posterior compartment (**I**), the boundary condition of lower end of the fracture line (**J**). *Note* a = anterior, p = posterior, m = medial, l = lateral

The modeling process is described below using the CAD model of the posteromedial tibial plateau fracture as an example to illustrate the modeling process. In this study, the cortical bone thickness was obtained by directly measuring the cortical bone thickness behind the tibial plateau of the bone specimen using mimic software (Mimics Medical 20.0). The 0.5 mm thick cortical bone was simulated with a surface element, and the cancellous bone was simulated with a solid element. The plates and screws were simulated using solid elements. Osteotomy of the bone in different directions was intended to simulate a typical fracture model; osteotomy along the edge of the tibia from the anterior with a width of 1 mm was used to simulate the fracture line. The cutting line was 90 °when the tibia was half the diameter. The angle was turned to the medial side of the tibial plateau until it was completely removed. The distal end of the fragment was then cut completely to separate it from the tibial plateau (Fig. 1A and B); and Fig. 1C shows the inner and outer structures of the bone. A tetrahedral element was used to simulate the plate. The plate and nail cap were tied to simulate the locking system, meaning that relative movement between the screws and plates was not possible, simulating the locking state (Fig. 1D and E). Details regarding meshing, including mesh size and the interaction used in this simulation, are presented in Table 2 [15]. The interactions used in this simulation, such as the interaction between bone and bone (fracture and residual bone), as well as between bone and screw, were referenced as follows: friction coefficient between bone and bone: 0.15; bone and screw: tie contact (binding between bone and screw without relative displacement).

**Table 2:** Model mesh size and mesh number

**Mesh size(mm)**	Screws	0.25
	Plate	0.25
	Cancellous bone	Nail-bone interface: 0.25,other 1
	Cortical bone	Nail-bone interface: 0.25,other 1
**Mesh number(n)**	Screws	284,838
	Plate	66,686
	Cancellous bone	1,506,744
	Cortical bone	37,975

To save computational resources, screw threads were simplified using a hexahedral mesh. Two other fracture fixation modes were established in the same manner (Fig. 1F and G).

**Fig. 2:**
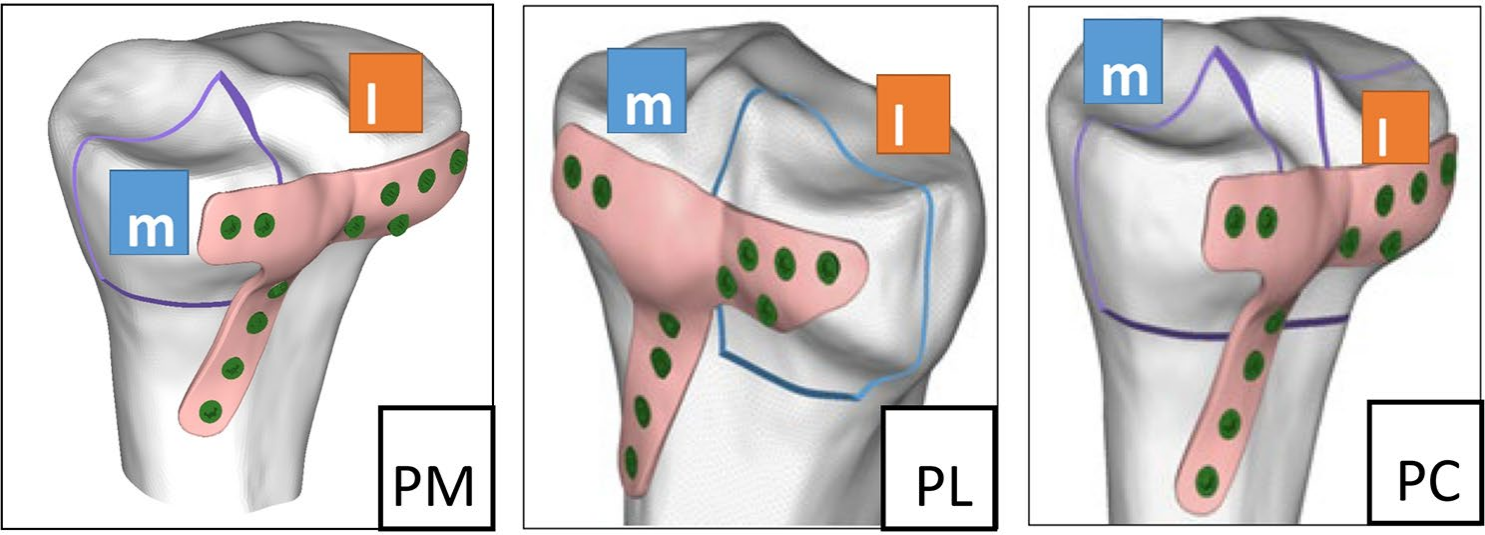
CAD model of the tibial plateau fracture indicates three groups:group PM (PM),group PL (PL),group PC (PC)

The model diagrams for the PM, PL and PC groups are shown in Fig. 2.

### Finite element analysis

Two loading methods were used in the experiment. The first is the uniform loading of the entire plateau. This was the load that the tibial plateau was subjected to during standing, walking, running, and jumping. The other was the uniform loading of the posterior plateau, which simulated tibia leveling of the knee joint when the knee was flexed. Under uniform loading on the plateau, the loading force was twice the standard body force applied by the average Chinese population (1500 N) [16]. In some finite element experiments, 750 N pressure was used as the biomechanical strength of the internal fixation for the stress test [16–18]. Considering the individual differences and the body weight of some patients in clinical practice varying between 75 Kgf and 150 Kgf, in the current study, to better evaluate the biomechanical strength of the new titanium plate, the high limit of patient weight, 1500 N, was used as the stress standard. We hypothesized that the use of a 1500 N load could simulate the stress and the change of the model under the overload condition (2 times the weight), and also observe whether the plate has excellent biomechanical properties.

The whole plateau (Fig. 1H) and posterior plateau of Groups PM, PL, and PC were loaded uniformly from top to bottom (Fig. 1I), Fig. 1J shows the boundary condition of the lower end of the fracture line.

The evaluation criteria were as follows: (i) the displacement of fracture in groups PM, PL, and PC during loading; (ii) the maximum stress on the plate and screws in groups PM, PL, and PC during loading; (iii) the stress distribution of the fracture, plate, and screws in groups PM, PL, and PC during the loading process.

## Results

When we conducted a full plateau loading experiment, the maximum displacements of the fracture block in groups PM, PL, and PC were 0.275, 0.114, and 0.265 mm, respectively, and the maximum stresses on the plate were 118.131, 44.191, and 115.433 MPa, respectively, whereas the maximum stress on the screw was 166.731, 80.330, and 164.439 MPa, respectively.

**Fig. 3:**
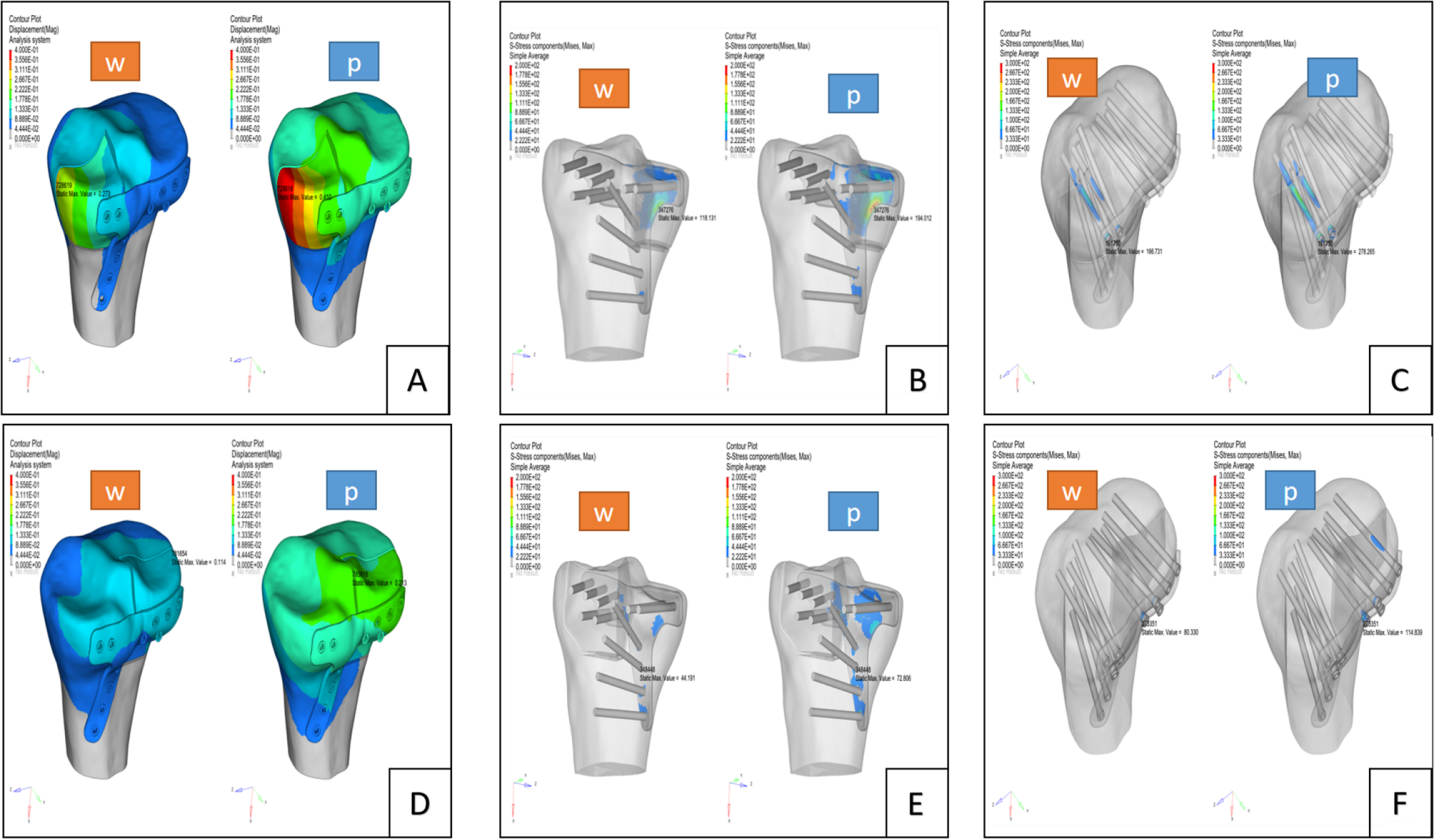
Finite element analysis showing fracture displacement, and analyzing the stress applied on plate and the stress on screws; group PM: fracture displacement (**A**), stress on plate (**B**), and stress on screws (**C**); in group PL: fracture displacement (**D**), stress on plate (**E**), and stress on screws (**F**) *Note* w = whole plateau was loaded, p = posterior plateau was loaded

**Fig. 4:**
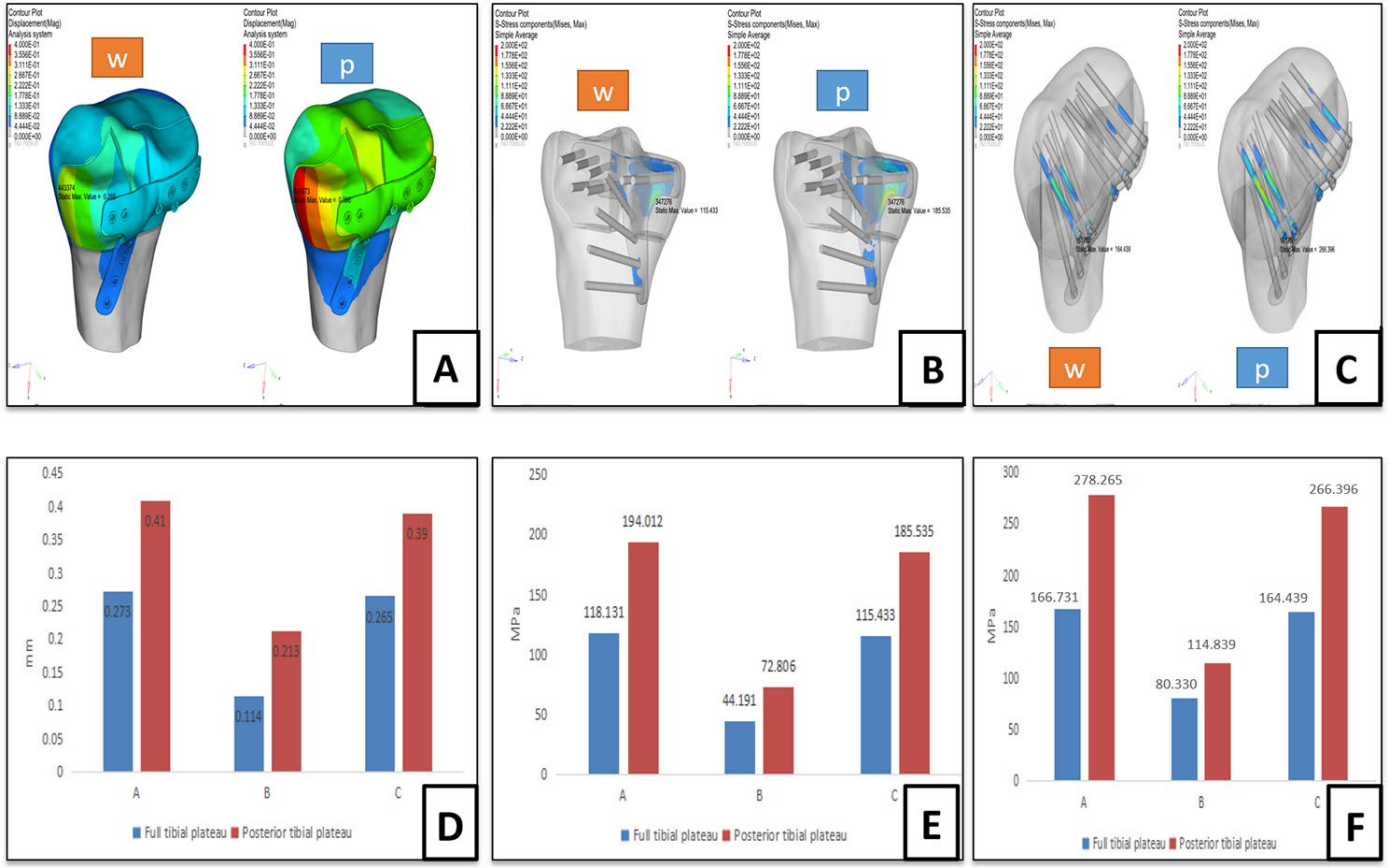
Finite element analysis showing fracture displacement, and analyzing the stress applied on the plate as well as the stress on the screws in group PC: fracture displacement (**A**), stress applied on the plate (**B**), and stress applied on the screws (**C**); graphical representation of the results on histogram:comparison of fracture displacement in the three groups (**D**), comparison of three groups of plates subjected to maximum stress (**E**) ,comparison of maximum stress on three sets of screws (**F**). *Note* w = whole plateau was loaded, p = posterior plateau was loaded

When we conducted the posterior plateau loading experiment, the maximum displacements of the fracture block in groups PM, PL, and PC were 0.410, 0.231, and 0.390 mm, respectively; the maximum stresses on the plate were 194.012, 72.806, and 185.535 MPa, respectively; and the maximum stresses on the screws were 278.265, 114.839, and 266.396 MPa, respectively. Figures 3 and 4 illustrate the above-mentioned findings, and the results of the finite element analysis are summarized in Table 3.

**Table 3:** Results of finite element analysis

Parameters		Group PM	Group PL	Group PC
**Whole plateau**	Displacement of fracture(mm)	0.273	0.114	0.265
	Maximum stress on plate(MPa)	118.131	44.191	115.433
	Maximum stress on screws(MPa)	166.731	80.330	164.439
**Posterior** **Plateau**	Displacement of fracture(mm)	0.410	0.213	0.390
	Maximum stress on plate(MPa)	194.012	72.806	185.535
	Maximum stress on screws(MPa)	278.265	114.839	266.396
**Analysis of stress on fracture block**		Medial side of medial tibial pateau fracture	Lateral condyle of tibial plateau and middle part between two tibial condyles	Medial side of medial tibial pateau fracture
**Analysis of stress on plate**		Medial side of proximal and distal plate junction	Medial side of proximal and distal plate junction, distal part of distal plate	Medial side of plate
**Analysis of stress on screws**		Head and body of two middle section screws	Head of three middle section screws	Head and body of middle screws

## Discussion

Simple posterior tibial plateau fractures such as Schatzker I can be effectively fixed with 2–3 lateral lag screws [19]; severe posterior tibial plateau fractures or fractures associated with other parts of the plateau require open reduction and internal fixation with plates and screws. Previously, the anterior approach was chosen, and the posterior fracture block was fixed using an anterolateral plate [6]. The advantage of this fixation method is that the surgical approach is simple and does not usually damage the posterior tibial neurovascular structures. It is easy for the surgeon to master; however, this fixation method cannot provide effective support in the posterior tibial plateau [20]. In early functional exercise, fracture displacement and plateau height loss were noticed [21]. Relevant clinical studies have shown that treatment of posterior tibial plateau fractures with a posterior support plate can provide a better therapeutic effect than other internal fixation methods [22]. With this method, the posterior support plate can be placed under direct vision at the fracture site. The reduction in the fracture is more satisfactory. The reduction of the articular surface is closer to anatomical reduction. The posterior buttress plate provided strong support for the tibial plateau [23]. The direction of the support is generally related to the orientation of the fracture, and the fixation effect is better. During the postoperative period, this can prevent fixation failure of the tibial plateau. Chen et al. [24] treated ten cases of posterior tibial plateau fractures with the posterior buttress plate; nine cases achieved anatomical reduction and one case resulted in a joint surface collapse of 2 mm. The follow-up knee score was 95.3 + 6.5 points and the knee flexion was 95–140 degrees.

Some authors believe that a simple lateral locking plate can provide adequate fixation strength in posterior tibial plateau fractures. Cho et al. [3] suggested the use of a proximal locking screw parallel to the coronal fracture line. The authors concluded that there was a mechanical loss of the cantilever load when the screws were fixed from the anterior side, and that with the use of an anterior plate, a strong and rigid fixation of the posterior tibial plateau could not be achieved. Fixation failure of the lag screw may lead to postoperative fracture displacement and traumatic arthritis complications. Sassoon et al. [25] found that an average distance of 16 mm was not fixed with a lateral locking plate in the treatment of posterior fractures. For these types of posterior fractures, the lateral plate cannot achieve an effective fixation.

The posterior buttress plate can be placed directly behind the tibial plateau; nevertheless, it is mandatory to pay special attention to the popliteal neurovascular bundle as well as the anterior tibial artery (as it arises from the popliteal artery in the popliteal fossa) during the placement of the plate. Even for surgeons familiar with the posterior approach, the plate should be placed under direct visualization to ensure vessel safety. The choice of placing the distal end plate slanting towards the medial side of the posterior tibial plateau is much safer. Zhang et al. [26] believed that for posterior fractures, the use of a double plate, that is, the posterior plate and anterior lateral plate, provides a stronger axial resistance for the posterior tibial plateau fracture and better maintenance of the articular surface. However, for a more complex tibial plateau fracture, fixation with the double plate is not completely reliable, and multiple plates are required to provide a stronger fixation.

Currently, no surgical approach or internal fixation is considered the gold standard for the treatment of posterior tibial plateau fractures. The posterior buttress and lateral plates are considered strong and reliable means of fixation [27]. Some authors have proposed ways to strengthen the fixation, such as hoop plates and tubular plates, which can provide effective and fixed support for the comminuted articular surface of the posterior tibial plateau fracture. Sun et al. [28] believed that the treatment of posterior tibial plateau fractures is related to surgical skills. However, the study of the fracture pattern in the posterior tibial plateau as well as whether a surgical approach or internal fixation is suitable for the same type of fracture is not much discussed; the authors used the self-made magic screw combined with a drifting plate for internal fixation through the lateral approach in the treatment of the posterior tibial plateau fracture; sixteen cases of the collapse posterior tibial fracture were treated with this method; the average range of motion (ROM) of knee reached 2.3–125 degrees in the last follow-up. The average HSS score of the joint was 94.2 points.

Posterior tibial plateau fractures often involve the articular surface; therefore, anatomical reduction must be performed. A vertical displacement of less than 2 mm was also considered a good reduction [29]. Good reduction or anatomical reduction is particularly important for the treatment of tibial plateau fractures. Early flexion and extension of the knee joint can be performed after internal fixation, but weight bearing of the affected limb should not be performed too early. Complete weight bearing at approximately 12 weeks was recommended.

The new plate designed in this experiment was sickle-shaped with a thickness of 2 mm, which usually relieves the discomfort of the plate in the thick muscles located behind the tibial plateau by decreasing the pressure of the plate over the soft tissues. Its advantage is that it maintains an adequate distance between the tibial plateau and tip of the fibula head. Therefore, an extra screw can be added to the locking hole of the new plate to strengthen the fixation effect on the lateral fracture. Although the new plate is used especially for the treatment of lateral tibial plateau fractures, patients with simple medial tibial plateau fractures can be treated simultaneously. The design of the distal plate extension to the medial side along the soleus muscle reduces interference with the anterior tibial vessel.

During this experiment, a pressure of 1500 N was applied to the entire tibial plateau or the posterior tibial plateau in groups PM, PL, and PC. The results showed that the degree of fracture displacement and the maximum stress of the plate were less in groups PM and PC than in group PL, indicating that the new plate was better for the treatment of posterolateral tibial plateau fractures than posteromedial and posterior fractures. Before the finite element analysis of the posterolateral tibial fracture using a plate, the loading force was equal to the physical quality of the human body, that is, 70 kgf was used as the standard, which was converted to the corresponding load. However, the failure of internal fixation of fractures is mostly due to long-term fatigue of the plate and screw. We believe that the load converted from the weight of ordinary people is insufficient to verify the reliability of our new titanium plate.

**Fig. 5:**
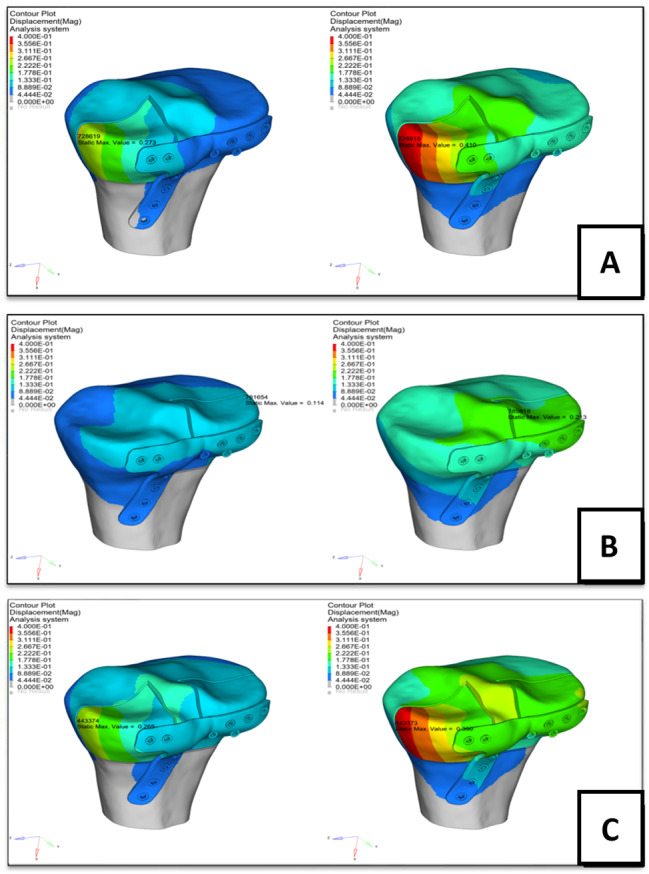
Diagram showing the stress distribution on the tibial plateau: stress distribution diagram of fracture block in group PM (**A**), stress distribution diagram of fracture block in group PL (**B**); fracture block distribution map in Group PC (**C**)

The distribution of stress in the plate and screw, as well as the fracture displacement, is similar in the single group even with different loading modes, but different between the groups in the same loading mode. Therefore, the stress distribution of the tibial plateau depended on the fracture type. The stress of the fracture on the medial and lateral sides of the tibial plateau was larger than that on the contralateral side **(**Fig. 5**)**. It is considered that the two sides of the tibial plateau have the greatest weight during the process of internal fixation; therefore, the surgeon can consciously strengthen the fixation of the two sides during the operation. Under the same loading conditions, the fracture displacement produced by posterior loading was greater than that of the entire loading. This shows that in a simple posterior plateau force, the fracture and plate are subjected to greater stress. The time required for patients to initiate a squatting position should be delayed to avoid failure of internal fixation caused by early squatting. The main mechanism of the posterolateral tibial plateau fracture is the axial force of the knee joint flexion leading to the femoral condyle impacting the tibial plateau, which is in accordance with the results of this experimental observation proposed in previous studies [3]. In groups PM, PL, and PC, there was uniformity in terms of stress distribution, resulting in a lower concentration of stress on the plate and screw; therefore, the possibility of plate fatigue, leading to internal fixation failure, was decreased.

Additionally, this new plate is expected to use the material from a traditional titanium alloy plate, which is currently widely used in clinical practice, and its strength is sufficient to meet the requirements of reduction and rigid fixation of fractures. The yield strength of titanium is 817 MPa [30, 31], and the maximum stress of the new plate and screw in this test is 278.265 MPa, which is far below its yield strength. We believe that this newly designed plate can be considered a potential alternative for the treatment of posterolateral tibial plateau fractures. At the same time, the displacement of the fracture in the experiment was not significant, which could improve bone growth [32, 33], but also avoid fracture nonunion associated with the relative movement of the fractured block [34].

This study is not without limitations. First, this study only tested the strength of the new titanium plate in the finite element analysis experiment and did not conduct a comparison with the conventional T-shaped steel plate. Second, special attention was not paid to the lower border of the simulated fracture line in the experimental model to compare the findings by considering the fracture shape and orientation, and the study did not contain clinical data demonstrating the biomechanical efficacy of this newly designed plate, which could open a new research field in the future.

## Conclusion

In this experiment, the finite element analysis of the new plate we developed suggested that it has good biomechanical efficacy in the treatment of posterolateral tibial plateau fractures. The results revealed a limited stress concentration on the plate and screw, minimized fracture displacement, and demonstrated the safety and effectiveness of fracture fixation.

## Data Availability

The datasets used and/or analyzed during the current study are available from the corresponding author upon reasonable request.

## Abbreviations

CAD: Computer Aided Design
ROM: Range of motion
